# Risk factors of acute ischemic stroke and the role of angiotensin I in predicting prognosis of patients undergoing endovascular thrombectomy

**DOI:** 10.3389/fendo.2024.1388871

**Published:** 2024-06-11

**Authors:** Shengkai Yang, Kemian Li, Zhengqian Huang, Yingda Xu, Jingshan Liang, Yong Sun, Aimin Li

**Affiliations:** ^1^ Department of Neurosurgery, The Affiliated Lianyungang Hospital of Xuzhou Medical University, Lianyungang, Jiangsu, China; ^2^ Department of Neurosurgery, Binhai County People’s Hospital Affiliated to Kangda College of Nanjing Medical University, Yancheng, Jiangsu, China

**Keywords:** acute ischemic stroke, risk factors, endovascular thrombectomy, angiotensin I, nomograms

## Abstract

**Purpose:**

The interaction between the renin-angiotensin system (RAS) and the acute ischemic stroke (AIS) is definite but not fully understood. This study aimed to analyze the risk factors of AIS and explore the role of serum indicators such as angiotensin I (Ang I) in the prognosis of patients undergoing endovascular thrombectomy (EVT).

**Patients and methods:**

Patients with AIS who underwent EVT and healthy controls were retrospectively enrolled in this study, and the patients were divided into a good or a poor prognosis group. We compared Ang I, blood routine indexes, biochemical indexes, electrolyte indexes, and coagulation indexes between patients and controls. We used univariate and multivariate logistic regression analyses to evaluate possible risk factors for AIS and the prognosis of patients undergoing EVT. Independent risk factors for the prognosis of patients undergoing EVT were identified through multifactorial logistic regression analyses to construct diagnostic nomograms, further assessed by receiver operating characteristic curves (ROC).

**Results:**

Consistent with previous studies, advanced age, high blood glucose, high D-dimer, and high prothrombin activity are risk factors for AIS. In addition, Ang I levels are lower in AIS compared to the controls. The level of Ang I was higher in the good prognosis group. Furthermore, we developed a nomogram to evaluate its ability to predict the prognosis of AIS after EVT. The AUC value of the combined ROC model (Ang I and albumin-globulin ratio (AGR)) was 0.859.

**Conclusions:**

In conclusion, advanced age, high blood glucose, high D-dimer, and high prothrombin activity are risk factors for AIS. The combined Ang I and AGR model has a good predictive ability for the prognosis of AIS patients undergoing arterial thrombectomy.

## Introduction

Acute ischemic stroke (AIS) is a disorder of blood flow supply to brain tissue caused by various reasons, which is characterized by high morbidity, disability, and mortality (1). According to statistics, the number of deaths due to ischemic stroke (IS) in the world ranks first in the number of deaths from cardiovascular and cerebrovascular diseases. It is one of the third leading causes of death in the world. China is a country with a high incidence of stroke. The incidence of stroke was 170/100,000 and 620/100,000 in males and females, respectively, and the prevalence rate was 620/100,000.

There are many pathogenic factors and complex pathological mechanisms of AIS. At present, it is generally believed that the occurrence of AIS is caused by atherosclerosis. Oxidative stress and vascular endothelial dysfunction are the primary pathogenesis of atherosclerosis. In recent years, with the deepening of research, people have found that the renin-angiotensin system (RAS) activation is closely related to AIS, which can directly or indirectly induce the occurrence and influence the development of AIS.

RAS is an endocrine regulatory system composed of peptide hormones and corresponding enzymes as the main components. It comprises six parts: renin, angiotensinogen, angiotensin, angiotensin-converting enzyme, angiotensin receptor, and aldosterone. Renin is an acid-hydrolytic protease produced by the juxtaglomerular cells of the kidney. Sodium depletion, sympathetic excitation, and decreased renal blood flow stimulate the release of renin, which acts on the hepatic production of angiotensinogen to convert it to angiotensin I (Ang I). Ang I has no biological activity and is further hydrolyzed to angiotensin II (Ang II) by the action of angiotensin-converting enzyme I. Ang II can be further decomposed into angiotensin (1–7) and angiotensin III and angiotensin IV by the step of angiotensin-converting enzyme II and aminopeptidase A, respectively (2).

Over-activation of RAS can produce a series of pathophysiological effects. At present, it is believed that circulating RAS mainly acts through the following two axes: (1) angiotensin-converting enzyme 1-Ang II-AT1R axis: the binding of Ang I-IV to AT1R leads to vasoconstriction, tissue cell fibrosis, and oxidative stress; (2) Angiotensin-converting enzyme 2-Ang (1–7)-Mas receptor axis: The binding of angiotensin domain with AT2R and Ang (1–7) with Mas receptor can resist the pathological effects of AT1R, dilate blood vessels, anti-inflammation, anti-tissue cell fibrosis, reduce cell apoptosis, and have protective effects on heart, brain, kidney, blood vessels and other organs (2). Studies on the pathological mechanism of RAS causing AIS mainly focus on Ang II, and the relationship between other components of RAS and AIS is relatively few.

The preferred treatment for AIS is intravenous thrombolysis within the time window. Still, due to the strict time window, the proportion of patients who can benefit from it is relatively low, and the treatment effect is poor (3). In recent years, with the continuous development of various endovascular therapy (EVT) devices and techniques, EVT has shown a good application prospect in treating AIS. Some domestic and foreign AIS treatment guidelines (4) recommend EVT as the first choice of treatment when intravenous thrombolysis is contraindicated or ineffective. Studies have shown that the overexpression of inflammatory factors and abnormal secretion of neurohormones in the pathogenesis of AIS aggravates the degree of neurological impairment and affects the prognosis of patients (5).

At present, the content of clinical evaluation of the development and prognosis of AIS mainly includes serological indicators and imaging examinations, among which serological indicators have the advantages of being simple, fast, and highly accurate and have become an essential means to guide clinical diagnosis and treatment. The primary objective of this study is to analyze the risk factors related to the occurrence of AIS, and the secondary aim is to explore the role of serum indicators such as neurohormones, blood routine, liver and kidney function, electrolytes, and coagulation function in the prognosis of patients undergoing endovascular thrombectomy (EVT).

## Materials and methods

### Patients

In this study, the alpha value was 0.05, the beta value was 0.2, and the test power was 0.8. The difference of Ang I between the patients and the controls was expected to be about 1–3 ng/ml. According to the 1:1 ratio between the case group and the control group, about 33 patients should be enrolled. Ultimately, seventy-two patients with AIS who underwent EVT at the Neurosurgery department of the First People’s Hospital of Lianyungang from December 2022 to October 2023 were retrospectively enrolled in this study. At the same time, 60 subjects who underwent regular physical examinations during the same period were included, and a total of 132 cases were included. This study met the criteria outlined in the Declaration of Helsinki. The ethics committee approved it, and the institutional review board of the First People’s Hospital of Lianyungang (ethics numbers: SHSY-IECKY-4.0/18–68/01 and ZDKYSB077). Written informed consent was obtained from this study’s patients and their relatives.

### Inclusion and exclusion criteria

Inclusion criteria: (1). Age over 18 years old; (2). AIS diagnosed by imaging (CT and MRI); (3). Undergo EVT.

Exclusion criteria: (1). Cerebral hemorrhage was confirmed by imaging examination; (2). Patients who have received thrombolytic therapy; (3). Concurrent diagnosis of other malignant tumors that may seriously affect survival; (4). Accompanied with severe infectious diseases or liver and kidney dysfunction; (5). Severe bleeding tendency; (6). Patients with previous IS and severe motor dysfunction.

Based on the above criteria, 7 patients accept thrombolytic therapy, 2 patients had severe liver and kidney dysfunction, and 3 patients had IS previously and severe motor dysfunction. We excluded 12 patients, and 60 patients were finally included in this study. According to the above criteria, we divided the 120 subjects into two groups: normal group and disease group. To further analyze the effect of different variables on the prognosis of patients with AIS who underwent EVT, 60 patients in the disease group were divided into good prognosis group (0–2 scores) and poor prognosis group (3–5 scores) according to the modified Rankin scale score at discharge.

### Study variables

The clinical data of inpatients in the Department of Neurosurgery of the First People’s Hospital of Lianyungang were collected retrospectively, including age, sex, blood routine indexes, biochemical indexes, electrolyte indexes, and coagulation indexes. Blood samples were collected within 72 hours after surgery for further analysis. All blood samples were obtained from either the left or right femoral vein. Rapidlab 1200 series equipment (Laboratory equipment of the First People’s Hospital of Lianyungang City) was used to analyze blood samples. Ang I ELISA assay: To measure the concentration of Ang I, quantitative factor high-sensitivity ELISA [R&D; Human angiotensin: BY-EH111540 (sensitivity 0.1 ng/mL)] was used to detect the concentration of Ang I in serum. Patients who underwent EVT were followed up by outpatient examination or telephone.

### Surgical methods of endovascular thrombectomy

The patient was conventionally given local anesthesia, and if the patient has agitation, general anesthesia or intravenous combined anesthesia will be selected. The patient was asked to take the supine position, the femoral artery puncture was performed, and the 8F artery sheath was inserted. The whole brain digital subtraction angiography (DSA) was performed to determine the infarction site. For patients without large artery occlusion, 100,000 U urokinase was injected into the artery on the opposite side of the lesion or the poor development side. Then 20, 000 U/min urokinase was maintained according to the patient’s condition. The vital signs of the patients were closely monitored. For patients with large artery occlusion, an 8F MPA1 guide catheter was placed at the distal end of the common carotid artery, and a 5F-125 Naven intermediate catheter was placed at the distal end of the internal carotid artery along the guide catheter. Under the guidance of a 0.014 microguide wire, a Rebar-18 microcatheter was used to pass through the occlusion vessel to the distal end of the thrombus. Microcatheter angiography was performed to confirm the patency of the thrombus site and the distal end of the occluded vessel. The Solitaire AB stent was placed through the microcatheter. After 5 minutes, to ensure the full release of the stent, the stent was withdrawn with the microcatheter, and the blood was quickly aspirated with a 50 mL syringe. DSA examination was performed again after thrombectomy to confirm vascular recanalization.

### Statistical analysis

Customarily distributed values were calculated as parametric tests and mean ± standard deviation, while non-normally distributed values were calculated as median. Categorical variables were analyzed by chi-square or Fisher’s exact test, and continuous variables were analyzed by unpaired t-test. The chi-square or Kruskal-Wallis test was used to evaluate the correlation between Ang I, blood routine indexes, biochemical indexes, electrolyte indexes, coagulation indexes, and clinicopathological features. The ROC curve and the area under the curve (AUC) were used to compare the ability of the models to predict AIS and outcome status. Univariate and multivariate logistic regression analyses were used to analyze the risk factors of AIS. Multivariate logistic regression was used to analyze the independent risk factors for the prognosis of AIS, and a diagnostic nomogram was constructed. All data were analyzed by SPSS software (version 27.0), GraphPad Prism software (version 8.3.1), and RStudio software (4.3.0). A value of 0.05 was considered statistically significant.

## Results

### Demographic and clinical characteristics

**Figure 1:**
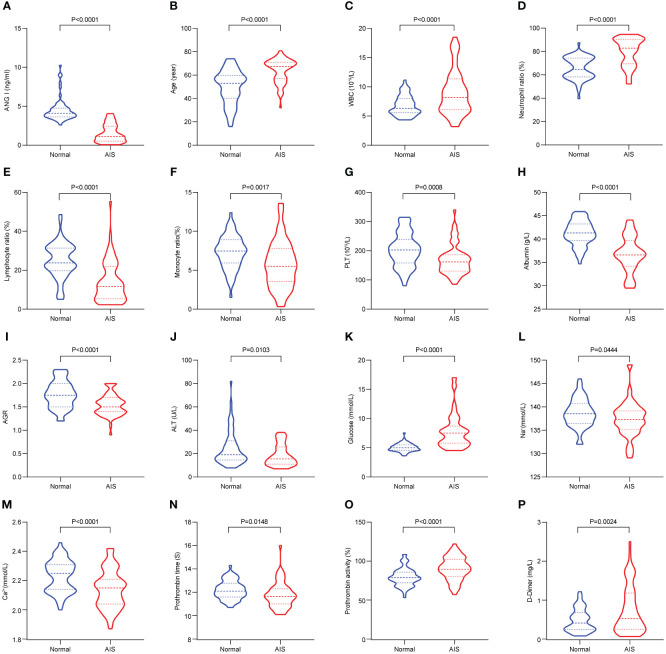
The significant differences in demographic data and clinical information between the controls and AIS patients. **(A)** Comparison of Ang I. **(B)** Comparison of age. **(C)** Comparison of WBC. **(D)** Comparison of neutrophil ratio. **(E)** Comparison of lymphocyte ratio. **(F)** Comparison of monocyte ratio. **(G)** Comparison of PLT. **(H)** Comparison of albumin. **(I)** Comparison of AGR. **(J)** Comparison of ALT. **(K)** Comparison of globulin. **(L)** Comparison of Na+. **(M)** Comparison of Ca2+. **(N)** Comparison of prothrombin time. **(O)** Comparison of prothrombin activity. **(P)** Comparison of D-Dimer. AIS, acute ischemic stroke; Ang I, angiotensin I; WBC, white blood cell; PLT, platelets; AGR, albumin-globulin ratio; ALT, alanine aminotransferase.

**Figure 1**; **Table 1** show the demographic data and various clinical information data between the controls and disease groups included in the study and the levels of significance of differences between groups. The median age of 120 patients was 56.925 ± 14.282 years old. The median age of 60 controls was 49.883 ± 14.418 years old, and the median age of 60 patients was 63.967 ± 10.133 years old (*p*<0.001). The level of Ang I in the disease group (1.52 ± 1.141) was significantly lower than that in the control group (4.531 ± 1.487) (*p*<0.001). WBC, neutrophil ratio, lymphocyte ratio, monocyte ratio, PLT in blood routine indexes; albumin, albumin-globulin ratio (AGR), ALT, glucose in biochemical indexes; K+, Ca2+ in electrolyte; prothrombin time, prothrombin activity and D-dimer all had significant differences between the normal group and the disease group (*p*<0.05).

**Table 1:** Baseline demographic and clinical characteristics of AIS patients and controls.

Characteristic	Total (n=120)	Controls (n=60)	AIS patients (n=60)	*P value*
Age	56.925±14.282	49.883±14.418	63.967±10.133	< 0.001
Sex				0.099
Female	55 (45.833)	32 (53.333)	23 (38.333)	
Male	65 (54.167)	28 (46.667)	37 (61.667)	
Ang I (ng/ml)	3.026±2.007	4.531±1.487	1.52±1.141	< 0.001
Anticoagulation				0.347
No	109 (90.833)	56 (93.333)	53 (88.333)	
Yes	11 (9.167)	4 (6.667)	7 (11.667)	
Atrial fibrillation				0.127
No	100 (83.333)	54 (90)	48 (80)	
Yes	20 (16.667)	6 (10)	12 (20)	
RAS receptor blocker therapy				0.228
No	86 (71.667)	46 (76.667)	40 (66.667)	
Yes	34 (28.333)	14 (23.333)	20 (33.333)	
Blood routine indexes				
WBC (10^9/L)	7.882±3.123	6.796±1.687	8.968±3.798	< 0.001
RBC (10^12/L)	4.244±0.596	4.267±0.684	4.222±0.497	0.68
Hb (g/L)	127.092±15.068	125.6±15.192	128.583±14.922	0.28
HCT (%)	38.453±4.435	37.922±4.302	38.985±4.537	0.19
Neutrophil ratio (%)	72.61±12.922	65.657±9.612	79.563±12.086	< 0.001
Lymphocyte ratio (%)	19.473±11.161	24.987±8.919	13.96±10.482	< 0.001
Monocyte ratio (%)	6.514±2.719	7.283±2.144	5.745±3.019	0.002
PLT (10^9/L)	185.525±56.71	202.617±57.649	168.433±50.686	< 0.001
Biochemical indexes				
Albumin (g/L)	38.977±3.972	41.233±2.621	36.72±3.819	< 0.001
Globulin (g/L)	24.212±4.436	24.095±4.686	24.33±4.207	0.773
AGR (%)	1.653±0.284	1.763±0.291	1.542±0.231	< 0.001
ALT (U/L)	21.609±12.614	24.542±14.871	18.677±9.071	0.011
AST (U/L)	24.677±8.581	25.303±9.469	24.05±7.619	0.426
BUN (mmol/L)	5.139±1.767	5.15±1.357	5.129±2.111	0.947
Cr (umol/L)	68.265±19.563	65.483±15.683	71.047±22.588	0.12
Uric acid (umol/L)	292.733±81.525	293.817±73.876	291.65±89.136	0.885
Glucose (mmol/L)	6.452±2.564	5.056±0.691	7.848±2.97	< 0.001
Electrolyte indexes				
K+ (mmol/L)	3.802±0.361	3.802±0.284	3.803±0.428	0.982
Na+ (mmol/L)	138.031±3.481	138.668±3.044	137.393±3.787	0.044
Cl- (mmol/L)	104.177±3.22	104.315±3.07	104.04±3.384	0.642
Ca2+ (mmol/L)	2.185±0.127	2.23±0.108	2.14±0.13	< 0.001
Coagulation indexes				
Prothrombin time (s)	11.995±0.98	12.212±0.789	11.778±1.103	0.015
Prothrombin activity (%)	85.014±14.312	79.538±11.556	90.49±14.785	< 0.001
INR	1.048±0.095	1.061±0.096	1.036±0.094	0.147
Partial thromboplastin time (s)	27.007±4.896	26.257±3.091	27.757±6.136	0.094
Thrombin time (s)	17.652±4.736	17.795±1.442	17.508±6.567	0.742
Fibrinogen (g/L)	2.849±0.746	2.847±0.525	2.852±0.919	0.971
D-Dimer (mg/L)	0.615±0.497	0.479±0.293	0.751±0.612	0.003

AIS, acute ischemic stroke; Ang I, angiotensin I; WBC, white blood cell; RBC, red blood cell; HCT, hematocrit; PLT, platelets; AGR, albumin-globulin ratio; ALT, alanine aminotransferase; AST, aspartate transaminase; BUN, blood urea nitrogen; INR, international normalized ratio.

### Risk factors for AIS

We explored the influencing factors of AIS occurrence by univariate and multivariate Logistic regression analyses (**Table 2**). Multivariate Logistic regression analysis showed that older age (OR: 1.098, 95%CI: 1.057–1.141, *p*<0.001), high leukocyte expression (OR: 1.327, 95%CI: 1.131–1.557, *p*<0.001), high neutrophil ratio (OR: 1.114, 95%CI: 1.070–1.160, p<0.001), hyperglycemia (OR: 4.512, 95%CI: 2.454–8.295, *p*<0.001), high prothrombin activity (OR: 1.064, 95%CI: 1.032–1.097, *p*<0.001), high D-dimer (OR: 3.515, 95%CI: 1.480–8.351, *p*=0.004) might be more likely to have AIS.

**Table 2:** Univariate and multivariate analysis of AIS patients and controls.

Characteristic	Univariate analysis	Multivariate analysis		
	Odds Ratio (95% CI)	*P value*	Odds Ratio (95% CI)	*P value*
Age	1.098(1.057-1.141)	<0.001	1.137(0.99-1.306)	0.07
Ang I (ng/ml)	0.048(0.013-0.184)	<0.001	0.019(0.001-0.266)	0.003
Blood routine indexes				
WBC (10^9/L)	1.327(1.131-1.557)	<0.001	–	0.764
RBC (10^12/L)	0.879(0.480-1.612)	0.677		
Hb (g/L)	1.013(0.989-1.038)	0.278		
HCT (%)	1.057(0.973-1.148)	0.190		
Neutrophil ratio (%)	1.114(1.070-1.160)	<0.001	–	0.764
Lymphocyte ratio (%)	0.892(0.852-0.934)	<0.001	–	0.484
Monocyte ratio (%)	0.797(0.687-0.925)	0.003	–	0.514
PLT (10^9/L)	0.988(0.981-0.996)	0.002	–	0.406
Biochemical indexes				
Albumin (g/L)	0.652(0.556-0.764)	<0.001	0.614(0.391-0.964)	0.034
Globulin (g/L)	1.012(0.933-1.098)	0.771		
AGR (%)	0.039(0.008-0.193)	<0.001	–	0.384
ALT (U/L)	0.959(0.927-0.992)	0.015	–	0.881
AST (U/L)	0.983(0.942-1.025)	0.423		
BUN (mmol/L)	0.993(0.810-1.217)	0.947		
Cr (umol/L)	1.015(0.996-1.036)	0.125		
Uric acid (umol/L)	1.000(0.995-1.004)	0.884		
Glucose (mmol/L)	4.512(2.454-8.295)	<0.001	4.731(0.712-31.448)	0.108
Electrolyte indexes				
K+ (mmol/L)	1.012(0.374-2.734)	0.982		
Na+ (mmol/L)	0.895(0.801-0.999)	0.049	–	0.606
Cl- (mmol/L)	0.974(0.871-1.089)	0.639		
Ca2+ (mmol/L)	0.002(0.000-0.054)	<0.001	–	0.729
Coagulation indexes				
Prothrombin time (s)	0.614(0.410-0.920)	0.018	–	0.304
Prothrombin activity (%)	1.064(1.032-1.097)	<0.001	–	0.103
INR	0.055(0.001-2.890)	0.151		
Partial thromboplastin time (s)	1.082(0.981-1.193)	0.113		
Thrombin time (s)	0.987(0.914-1.066)	0.740		
Fibrinogen (g/L)	1.009(0.623-1.634)	0.970		
D-Dimer (mg/L)	3.515(1.480-8.351)	0.004	–	0.246

AIS, acute ischemic stroke; Ang I, angiotensin I; WBC, white blood cell; RBC, red blood cell; HCT, hematocrit; PLT, platelets; AGR, albumin-globulin ratio; ALT, alanine aminotransferase; AST, aspartate transaminase; BUN, blood urea nitrogen; INR, international normalized ratio.

The higher Ang I (OR: 0.048, 95%CI: 0.013–0.184, *p*<0.001), the higher lymphocyte ratio (OR: 0.892, 95%CI: 0.852–0.934, *p*<0.001), higher monocyte ratio (OR: 0.797, 95%CI: 0.687–0.925, *p*=0.003), higher PLT (OR: 0.988, 95%CI: 0.981–0.996, *p*=0.002), and higher albumin (OR: 0.797, 95%CI: 0.687–0.925, *p*=0.002). 0.652, 95%CI 0.556–0.764, *p*<0.001), AGR (OR: 0.039, 95%CI: 0.008–0.193, *p*=0.049), ALT (OR: 0.959, 95%CI: 0.927–0.992, *p*=0.015), and Na+ (OR: 0.959, 95%CI: 0.927–0.992, *p*=0.015). 0.895, 95%CI = 0.801–0.999, *p*=0.049), and the higher Ca2+ (OR: 0.002, 95%CI = 0.000–0.054, *p*<0.001) and longer prothrombin time (OR<: 0.614, 95%CI: 0.410–0.920, *p*=0.018) might reduce the risk of AIS.

### Demographic and clinical characteristics of patients with different prognosis

**Table 3**; **Figure 2** show the demographic data and various clinical information data between the good and poor prognosis groups of AIS patients and the levels of significance of differences between groups. The median age of 60 patients was 63.967 ± 10.133 years old. The median age of 25 patients in the good prognosis group was 58.24 ± 10.887 years old, and the median age of 35 patients in the poor prognosis group was 68.057 ± 7.292 years old (*p*<0.001). The level of Ang I was significantly different between the good prognosis group (2.205 ± 1.256) and the poor prognosis group (1.031 ± 0.745) (*p*<0.001). Significant differences existed in the expression of globulin, AGR, ALT, K+, and prothrombin activity between the good and poor prognoses groups (*p*<0.05).

**Table 3:** Comparison of good prognosis and poor prognosis in AIS patients.

Characteristic	Total (n=60)	Good prognosis (n=25)	Poor prognosis (n=35)	P value
Age	63.967±10.133	58.24±10.887	68.057±7.292	< 0.001
Sex				0.164
Female	23 (38.333)	7 (28)	16 (45.714)	
Male	37 (61.667)	18 (72)	19 (54.286)	
Ang I (ng/ml)	1.52±1.141	2.205±1.256	1.031±0.745	< 0.001
Blood routine indexes				
WBC (10^9/L)	8.968±3.798	8.612±2.343	9.222±4.583	0.504
RBC (10^12/L)	4.222±0.497	4.239±0.402	4.21±0.56	0.816
Hb (g/L)	128.583±14.922	130.96±16.349	126.886±13.805	0.316
HCT (%)	38.985±4.537	39.72±4.57	38.46±4.506	0.295
Neutrophil ratio (%)	79.563±12.086	78.512±11.196	80.314±12.791	0.565
Lymphocyte ratio (%)	13.96±10.482	15.868±11.538	12.597±9.598	0.252
Monocyte ratio (%)	5.745±3.019	5.385±2.896	6.003±3.12	0.434
PLT (10^9/L)	168.433±50.686	172.8±48.267	165.314±52.816	0.571
Biochemical indexes				
Albumin (g/L)	36.72±3.819	36.748±3.481	36.7±4.094	0.961
Globulin (g/L)	24.33±4.207	23±2.992	25.28±4.708	0.026
AGR (%)	1.54±0.23	1.62±0.23	1.48±0.21	0.018
ALT (U/L)	18.677±9.071	18.608±9.039	18.726±9.226	0.961
AST (U/L)	24.05±7.619	21.04±6.248	26.2±7.858	0.006
BUN (mmol/L)	5.129±2.111	4.858±2.242	5.322±2.023	0.415
Cr (umol/L)	71.047±22.588	69.84±24.781	71.909±21.214	0.737
Uric acid (umol/L)	291.65±89.136	302.176±73.593	284.131±99.119	0.422
Glucose (mmol/L)	7.848±2.97	7.521±2.891	8.081±3.045	0.472
Electrolyte indexes				
K+ (mmol/L)	3.803±0.428	3.992±0.337	3.668±0.438	0.002
Na+ (mmol/L)	137.393±3.787	137.632±2.255	137.223±4.608	0.651
Cl- (mmol/L)	104.04±3.384	104.148±3.603	103.963±3.27	0.839
Ca2+ (mmol/L)	2.14±0.13	2.143±0.133	2.139±0.13	0.909
Coagulation indexes				
Prothrombin time (s)	11.778±1.103	11.496±1.241	11.98±0.962	0.11
Prothrombin activity (%)	90.49±14.785	96.064±14.444	86.509±13.888	0.013
INR	1.036±0.094	1.025±0.109	1.043±0.083	0.484
Partial thromboplastin time (s)	27.757±6.136	29.352±8.704	26.617±2.941	0.142
Thrombin time (s)	17.508±6.567	19.056±9.626	16.403±2.533	0.19
Fibrinogen (g/L)	2.852±0.919	2.654±0.683	2.993±1.043	0.134
D-Dimer (mg/L)	0.751±0.612	0.717±0.67	0.776±0.576	0.726

AIS, acute ischemic stroke; Ang I, angiotensin I; WBC, white blood cell; RBC, red blood cell; HCT, hematocrit; PLT, platelets; AGR, albumin-globulin ratio; ALT, alanine aminotransferase; AST, aspartate transaminase; BUN, blood urea nitrogen; INR, international normalized ratio.

**Figure 2:**
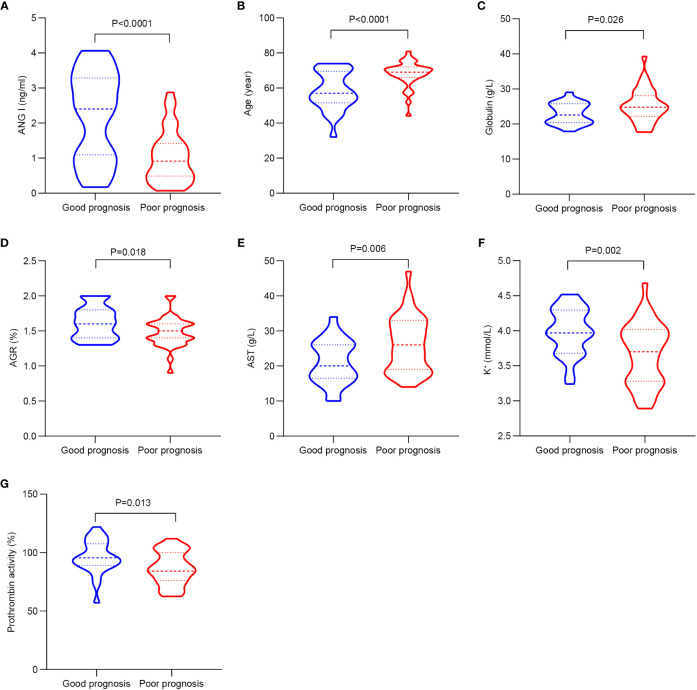
The significant differences in demographic data and clinical information between the good and poor prognosis groups of AIS patients. **(A)** Comparison of Ang I. **(B)** Comparison of age. **(C)** Comparison of globulin. **(D)** Comparison of AGR. **(E)** Comparison of AST. **(F)** Comparison of K+. **(G)** Comparison of prothrombin activity. AIS, acute ischemic stroke; Ang I, angiotensin I; AGR, albumin-globulin ratio; AST, aspartate transaminase.

### Identification of prognostic factors for AIS

**Figure 3:**
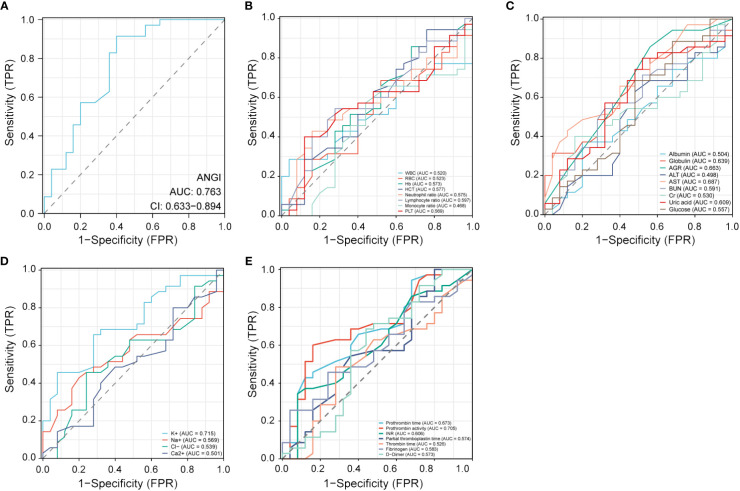
Receiver operating characteristic (ROC) curve analysis of different factors for the prognosis of AIS. **(A)** Ang I. **(B)** Blood routine indexes. **(C)** Biochemical indexes. **(D)** Electrolyte indexes. **(E)** Coagulation indexes. AIS, acute ischemic stroke; Ang I, angiotensin I; WBC, white blood cell; RBC, red blood cell; HCT, hematocrit; PLT, platelets; AGR, albumin-globulin ratio; ALT, alanine aminotransferase; AST, aspartate transaminase; BUN, blood urea nitrogen; INR, international normalized ratio.

To further explore the clinical diagnostic predictive value of serum Ang I, blood routine indexes, biochemical indexes, electrolyte indexes, and coagulation indexes for the prognosis of AIS, the good prognosis group was used as negative samples, and the poor prognosis group was used as positive samples. The ROC curve diagnostic analysis model was established. Ang I, K+, and prothrombin activity all had high diagnostic and predictive value. The AUC of Ang I was 0.763, 95%CI was 0.633–0.894, *p*<0.001; The AUC of K+ in electrolyte was 0.715, 95%CI was 0.585–0.846, *p*=0.005; The AUC of prothrombin activity in coagulation function was 0.705, 95%CI was 0.568–0.841, *p*=0.007 (**Table 4**; **Figure 3**).

**Table 4:** AUC of different factors for AIS.

Characteristic	AUC	Odds Ratio (95% CI)	P value
Ang I (ng/ml)	0.763	0.633-0.894	0.001
Blood routine indexes			
WBC (10^9/L)	0.520	0.372-0.668	0.793
RBC (10^12/L)	0.523	0.373-0.673	0.759
Hb (g/L)	0.573	0.421-0.725	0.341
HCT (%)	0.577	0.426-0.728	0.311
Neutrophil ratio (%)	0.575	0.426-0.723	0.326
Lymphocyte ratio (%)	0.597	0.449-0.744	0.205
Monocyte ratio (%)	0.468	0.314-0.622	0.675
PLT (10^9/L)	0.569	0.419-0.719	0.364
Biochemical indexes			
Albumin (g/L)	0.504	0.354-0.654	0.958
Globulin (g/L)	0.639	0.498-0.781	0.067
AGR (%)	0.663	0.521-0.805	0.033
ALT (U/L)	0.498	0.344-0.652	0.982
AST (U/L)	0.687	0.553-0.822	0.014
BUN (mmol/L)	0.591	0.443-0.739	0.233
Cr (umol/L)	0.530	0.380-0.680	0.691
Uric acid (umol/L)	0.609	0.460-0.758	0.152
Glucose (mmol/L)	0.557	0.402-0.712	0.458
Electrolyte indexes			
K+ (mmol/L)	0.715	0.585-0.846	0.005
Na+ (mmol/L)	0.569	0.422-0.715	0.368
Cl- (mmol/L)	0.539	0.388-0.690	0.61
Ca2+ (mmol/L)	0.501	0.350-0.652	0.994
Coagulation indexes			
Prothrombin time (s)	0.673	0.532-0.813	0.024
Prothrombin activity (%)	0.705	0.568-0.841	0.007
INR	0.606	0.458-0.753	0.165
Partial thromboplastin time (s)	0.574	0.421-0.726	0.333
Thrombin time (s)	0.526	0.373-0.526	0.73
Fibrinogen (g/L)	0.583	0.437-0.729	0.277
D-Dimer (mg/L)	0.573	0.414-0.732	0.337

AIS, acute ischemic stroke; Ang I, angiotensin I; WBC, white blood cell; RBC, red blood cell; HCT, hematocrit; PLT, platelets; AGR, albumin-globulin ratio; ALT, alanine aminotransferase; AST, aspartate transaminase; BUN, blood urea nitrogen; INR, international normalized ratio.

### Factors associated with the prognosis of AIS

We explored the influencing factors of AIS outcome by univariate and multivariate logistic regression (**Table 5**). The results showed that the higher Ang I (OR: 0.336, 95%CI: 0.184–0.616, *p*<0.001), the higher lymphocyte ratio (OR: 0.892, 95%CI: 0.852–0.934, *p*<0.001), higher AGR (OR = 0.053, 95%CI: 0.004–0.687, *p*=0.025), higher K+ (OR = 0.124, 95%CI: 0.028–0.555, *p*=0.006), and higher prothrombin activity (OR = 0.124, 95%CI: 0.028–0.555, *p*=0.006). 0.952, 95%CI: 0.914–0.991, *p*=0.017) might have a better prognosis.

**Table 5:** Univariate and multivariate analysis of good and poor prognosis AIS.

Characteristic	Univariate analysis		Multivariate analysis	
	Odds Ratio (95% CI)	*P value*	Odds Ratio (95% CI)	*P value*
Ang I (ng/ml)	0.336(0.184-0.616)	<0.001	0.26(0.124-0.547)	<0.001
Blood routine indexes				
WBC (10^9/L)	1.045(0.909-1.201)	0.538		
RBC (10^12/L)	0.887(0.312-2.520)	0.822		
Hb (g/L)	0.981(0.947-1.017)	0.297		
HCT (%)	0.939(0.836-1.055)	0.289		
Neutrophil ratio (%)	1.013(0.970-1.057)	0.567		
Lymphocyte ratio (%)	0.970(0.922-1.021)	0.240		
Monocyte ratio (%)	1.073(0.900-1.279)	0.433		
PLT (10^9/L)	0.997(0.987-1.007)	0.571		
Biochemical indexes				
Albumin (g/L)	0.997(0.870-1.141)	0.961		
Globulin (g/L)	1.164(1.004-1.349)	0.045	–	0.997
AGR (%)	0.053(0.004-0.687)	0.025	0.011(0.000-0.306)	0.008
ALT (U/L)	1.001(0.946-1.060)	0.960		
AST (U/L)	1.111(1.022-1.207)	0.013	–	0.081
BUN (mmol/L)	1.118(0.861-1.451)	0.403		
Cr (umol/L)	1.004(0.981-1.028)	0.725		
Uric acid (umol/L)	0.998(0.992-1.004)	0.439		
Glucose (mmol/L)	1.070(0.891-1.284)	0.471		
Electrolyte indexes				
K+ (mmol/L)	0.124(0.028-0.555)	0.006	–	0.263
Na+ (mmol/L)	0.971(0.847-1.114)	0.678		
Cl- (mmol/L)	0.984(0.844-1.147)	0.833		
Ca2+ (mmol/L)	0.789(0.015-42.147)	0.907		
Coagulation indexes				
Prothrombin time (s)	1.577(0.915-2.716)	0.101		
Prothrombin activity (%)	0.952(0.914-0.991)	0.017	–	0.106
INR	8.858(0.028-2822.600)	0.458		
Partial thromboplastin time (s)	0.910(0.805-1.029)	0.134		
Thrombin time (s)	0.933(0.846-1.029)	0.165		
Fibrinogen (g/L)	1.559(0.834-2.915)	0.164		
D-Dimer (mg/L)	1.174(0.499-2.759)	0.713		
Prothrombin time (s)	1.203(1.046-1.384)	0.010	–	0.194

AIS, acute ischemic stroke; Ang I, angiotensin I; WBC, white blood cell; RBC, red blood cell; HCT, hematocrit; PLT, platelets; AGR, albumin-globulin ratio; ALT, alanine aminotransferase; AST, aspartate transaminase; BUN, blood urea nitrogen; INR, international normalized ratio.

High expression of globulin (OR: 1.164, 95%CI: 1.004–1.349, *p*=0.045), increased expression of ALT (OR: 1.111, 95%CI: 1.022–1.207, *p*=0.013), and high admission NIHSS score (OR: 1.203, 95%CI: 1.046–1.384, *p*=0.010) might have a poor prognosis of AIS. Multivariate Logistic regression analysis of variables with significant differences in univariate logistic regression showed that Ang I (OR: 0.260, 95%CI: 0.124–0.547, *p*<0.001) and high AGR (OR: 0.011, 95%CI: 0.000–0.306, *p*=0.008) were still statistically significant.

### Nomogram construction and validation

**Figure 4:**
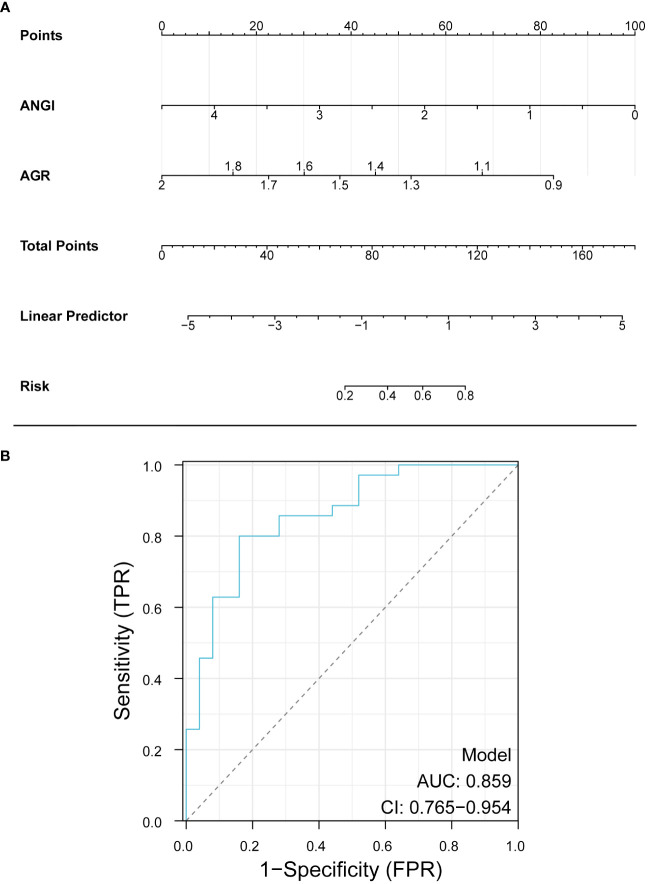
Nomogram for predicting the prognosis of AIS. Ang I, angiotensin I; AGR, albumin-globulin ratio.

According to the results of multivariate logistic regression, Ang I and AGR were selected to construct a diagnostic nomogram for the prognosis of AIS, and the ROC curve was used to verify the diagnostic nomogram to test its predictive efficacy for the prognosis of patients. The AUC value of the combined model was 0.859 (95%CI: 0.765–0.954). According to the above results, it was suggested that the model had a solid predictive ability, as shown in **Figure 4**.

## Discussion

In this retrospective study, univariate and multivariate logistic regression were used to analyze the risk factors for AIS. Consistent with previous studies, advanced age, high blood glucose, high D-dimer, and high prothrombin activity are risk factors for acute ischemic AIS. We also found that the increase of white blood cells and the increase of neutrophil ratio in blood routine examination also have specific suggestive significance. In addition, we found that the higher Ang I was, the lower the risk of AIS was. The level of Ang I was higher in the good prognosis group. Significant differences existed in the expression of globulin, AGR, ALT, K+, and prothrombin activity between the good and the poor prognosis group. As previously mentioned, our study found that Ang I and AGR had a non-negligible impact on the prognosis of patients with AIS, so we developed a nomogram to evaluate its ability to predict the prognosis of AIS.

Risk factors for AIS include age, smoking, obesity, atrial fibrillation, hypertension, diabetes, etc. (6), of which hypertension is the most critical risk factor. Excluding other risk factors for AIS, a 10mmHg increase in systolic blood pressure was associated with a 49% increased risk of AIS, and a 5mmHg rise in diastolic blood pressure was associated with a 46% increased risk of AIS (7). Experimental results showed that the vascular shear stress was increased in hypertensive patients compared with normal people, which caused vascular endothelial damage (8). At the same time, endothelial injury also affects NO activity and increases the synthesis and release of endothelin and Ang II, resulting in vasomotor dysfunction (8). Wallace et al. found that after endothelial injury, endothelial cells will release a large number of active substances, including adenosine triphosphate, 5-hydroxytryptamine, etc., which can promote the synthesis and release of endothelin, increasing blood pressure, and then damage endothelial cells again, forming a vicious circle (9). The primary pathogenesis of AIS is as follows: The damage of vascular endothelial cells leads to atherosclerosis, which leads to occlusion of cerebral arteries and cerebral ischemia. Neurons in the ischemic center release the injury-related molecular pattern to activate the immune response (10–12).

Hyperactivation of the local renin-angiotensin-aldosterone system (RAAS) in hypertensive patients is also an important reason for vascular endothelial injury (13). RAAS is an essential regulatory system to maintain the body’s balance of water and electrolyte. Its dysfunction will lead to vasoconstriction and aggravate the degree of ischemic injury in cerebrovascular diseases. And affect the rate of vascular recanalization after treatment (14, 15). The mechanism of RAAS activation is as follows: firstly, angiotensinogen is converted into Ang I under the stimulation of renin, and then Ang I is converted into Ang II by ACE. Ang II increases aldosterone secretion through the sympathetic nervous system, resulting in water and sodium retention and increasing blood pressure (16).

Some studies have shown that the serum Ang II level of patients after intravenous thrombolysis or EVT treatment is significantly lower than that before treatment, and the serum Ang II level of patients in the EVT group is lower than that in intravenous thrombolysis group, suggesting that EVT can significantly reduce the neurohormone level of AIS patients (17–22). How does the level of Ang I change? Our study focused on the changes of Ang I.

Our study found that Ang I levels were lower in AIS patients than in controls and higher in the group with a good prognosis. One study had similar results to ours. The level of Ang I decreased while the level of Ang II remained unchanged, accompanied by an increase in ACE activity. This phenomenon can be explained by the decrease in renin caused by the negative feedback after the rise in blood pressure. Increased ACE activity can convert all available Ang I to Ang II (16). Another reason for maintaining a steady Ang II concentration may be the up-regulation of local AT1 and AT2 receptors in and around the infarct area. Animal experiments have also confirmed that RAAS promotes brain oxidative stress response, further promoting RAAS and sympathetic nervous system activation to mobilize peripheral organ responses (23).

Nomograms are highly effective in areas such as diagnosis and prediction. Compared with traditional scoring systems for IS (24, 25), the nomogram model can estimate the probability of adverse outcomes after EVT, which often provides a better-personalized assessment to help management decisions. In addition, the graph has higher accuracy and better-discriminating ability and is more convenient to use. Several studies have predicted poor outcomes in patients with IS. Zhang et al. established a nomogram model to predict the 3-month risk of death in IS patients with anterior circulation arterial occlusion who successfully received endovascular thrombolysis, composed of age, pretreatment collateral status, baseline blood glucose level, symptomatic intracranial hemorrhage, and baseline National Institutes of Health Stroke Scale score (26). Du et al. determined that age, baseline National Institutes of Health Stroke Scale score, collateral circulation, rapid blood glucose levels, and recirculation were independent predictors of malignant cerebral edema after EVT (27). A study from Japan also focused on whether imaging technology, low relative diffusion-weighted imaging (DWI) signal intensity, can predict good clinical outcomes after EVT in patients with acute IS. Forty-nine patients were included in the analysis, and the results showed that the relative DWI signal intensity of the group with a good prognosis was significantly lower than that of the group with a poor prognosis. Low relative DWI signal intensity was associated with a good prognosis after EVT (28). In this study, Ang I and AGR, two readily available variables, have good discriminative ability, and the AUC value of the combined ROC model is 0.859. Therefore, the combined Ang I and AGR model can predict the prognosis of AIS patients, thus achieving a more accurate therapeutic effect and better serving the patient population.

The small sample size limits our study, and the sample size will be expanded. This is a retrospective single-center study, and the results must be further prospectively verified in multiple centers. Our study has established the role of Ang I in the prognosis of patients undergoing EVT. Still, there was no study of the other components, including angiotensinogen and Ang II, so further studies are needed to explore the mechanisms and other parts of the RAAS system.

## Conclusion

The study’s findings suggested advanced age, high blood glucose, high D-dimer, and high prothrombin activity are risk factors in patients with AIS. The higher the ANG I and AGR, the better the prognosis of AIS surgery. Furthermore, the combined ANG I and AGR model has a good predictive ability for the prognosis of patients undergoing arterial thrombectomy.

## Data availability statement

The original contributions presented in the study are included in the article/supplementary material. Further inquiries can be directed to the corresponding authors.

## Ethics statement

The studies involving humans were approved by The institutional review board of the First People’s Hospital of Lianyungang. The studies were conducted in accordance with the local legislation and institutional requirements. The participants provided their written informed consent to participate in this study.

## Author contributions

SY: Conceptualization, Data curation, Formal Analysis, Writing – original draft. KL: Methodology, Software, Visualization, Writing – review & editing. ZH: Validation, Writing – review & editing. YX: Validation, Writing – review & editing. JL: Validation, Writing – review & editing. YS: Funding acquisition, Investigation, Resources, Supervision, Writing – review & editing. AL: Conceptualization, Project administration, Supervision, Writing – review & editing.
